# Beyond the stomach: early bone metastases revealing canine gastric adenocarcinoma

**DOI:** 10.1111/jsap.70134

**Published:** 2026-04-16

**Authors:** G. d. S. Schiavi, C. Y. A. Kawabata, J. V. P. Campos, L. N. Lima, A. M. A. Abranches, L. L. Patente, V. D. Laranjeira, V. Nowosh, J. V. de Assis, A. J. S. de Souza

**Affiliations:** ^1^ Veterinary Clinic of UNISA Santo Amaro University (UNISA) São Paulo Brazil; ^2^ Postgraduate Program in One Health Santo Amaro University (UNISA) São Paulo Brazil; ^3^ Faculty of Veterinary Medicine Santo Amaro University (UNISA) São Paulo Brazil; ^4^ Department of Internal Medicine, School of Veterinary Medicine and Animal Science University of São Paulo (USP) São Paulo Brazil

A 9‐year‐old male shih‐tzu dog was admitted with a 4‐day history of lameness in the left pelvic limb, with no radiographic abnormalities. Abdominal ultrasonography revealed thickening of the gastric body wall and splenomegaly. The dog showed transient improvement in lameness after anti‐inflammatory therapy; however, 2 weeks later, it returned with severe pain and impairment of the left thoracic limb. Repeat radiographic evaluation demonstrated multiple osteolytic lesions (left femur, left ilium, fifth lumbar vertebra and left humerus) (Fig 1A). Fine‐needle aspiration cytology of the humerus suggested a malignant round cell neoplasm (Fig 1B). Eighteen days after admission, the animal showed clinical worsening, intense pain, and poor response to analgesic and supportive therapies. Given poor prognosis, euthanasia was elected and necropsy was performed. Grossly, the stomach showed mural thickening of the gastric body and pyloric antrum, forming ulcerated plaque‐like lesions (1.0 to 1.5 cm). The splenic parenchyma exhibited irregular white‐to‐yellow areas. Multifocal bone lysis was observed, and the mesenteric lymph nodes were enlarged. Tissue samples were collected for histopathology (haematoxylin and eosin and Alcian blue [AB]), and immunohistochemistry assessed pan‐cytokeratin (AE1/AE3) expression. Microscopically, a poorly demarcated, multifocal infiltrative neoplasm involved all layers of the stomach. The neoplasm exhibited an irregular tubular arrangement of AB‐positive polygonal to round cells with frequent signet‐ring morphology (Fig 1C). Frequent lymphovascular invasion and 13 mitotic figures in 2.37 mm^2^ were observed. Pan‐cytokeratin immunostaining confirmed the epithelial origin of the primary mixed‐type gastric adenocarcinoma and its metastases in the spleen, lymph nodes, liver and bones (Fig 1D–F).

**FIG 1:**
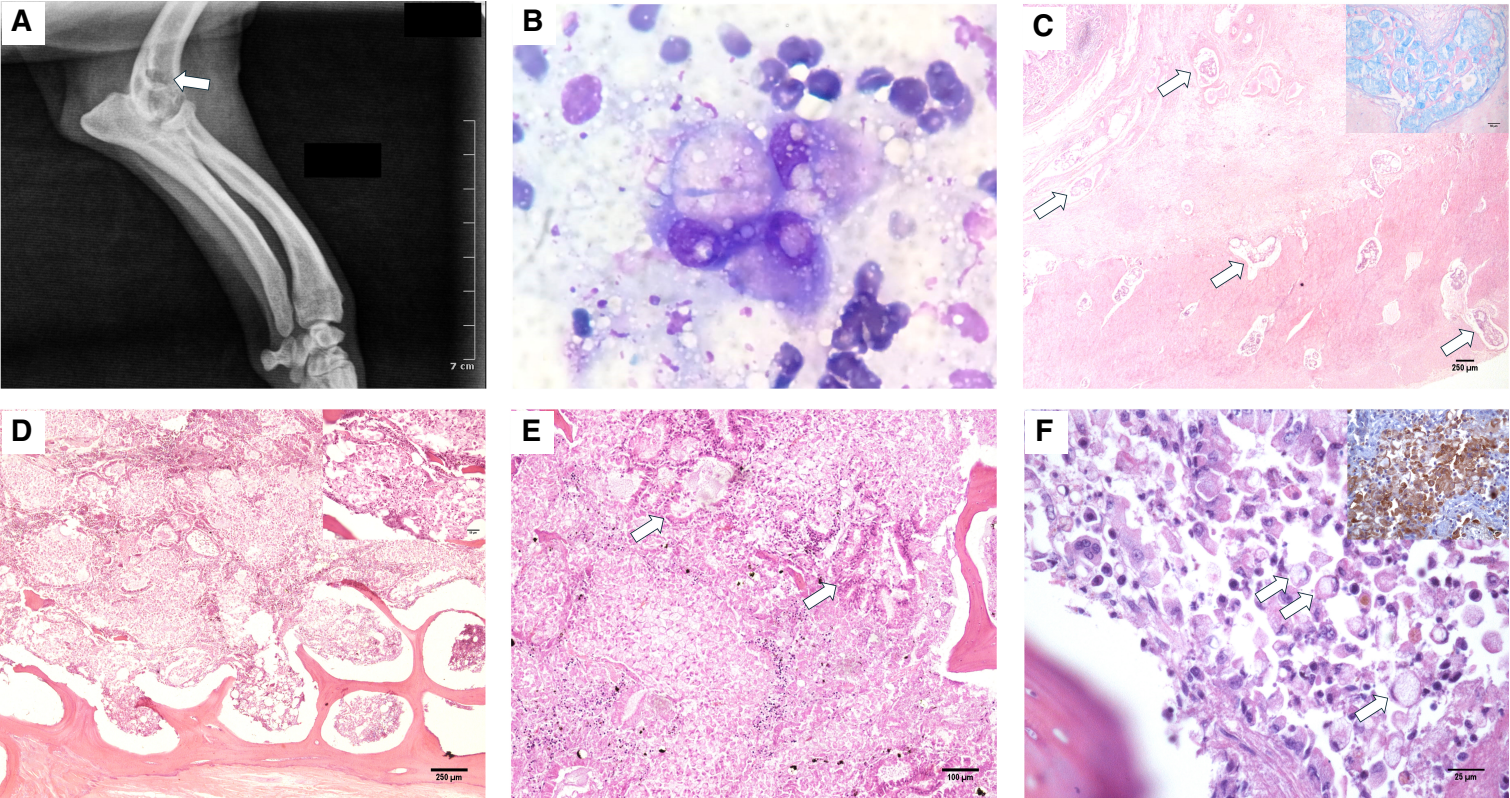
(A) Osteolysis (arrow). (B) Vacuolated epithelial cells, signet‐ring cell. (C) Stomach: infiltrative epithelial neoplasia (arrows); mucin (AB, inset). (D) Ilium: neoplastic cells effacing bone marrow. (E) Femur: neoplastic tubules (arrows). (F) Humeral bone marrow: signet‐ring cells; pan‐cytokeratin positivity (IHC, inset).

## Author contributions

**G. d. S. Schiavi:** Investigation; methodology; validation; visualization; writing – review and editing; formal analysis; data curation; writing – original draft. **C. Y. A. Kawabata:** Investigation; methodology; validation; visualization; writing – review and editing; formal analysis; data curation; writing – original draft. **J. V. P. Campos:** Investigation; writing – original draft; methodology; visualization; writing – review and editing; formal analysis. **L. N. Lima:** Investigation; writing – original draft; methodology; visualization; writing – review and editing; formal analysis. **A. M. A. Abranches:** Investigation; writing – original draft; methodology; visualization; writing – review and editing; formal analysis. **L. L. Patente:** Investigation; writing – original draft; methodology; visualization; writing – review and editing; formal analysis. **V. D. Laranjeira:** Investigation; writing – original draft; methodology; visualization; writing – review and editing; formal analysis. **V. Nowosh:** Investigation; writing – original draft; methodology; formal analysis; validation; visualization; writing – review and editing; data curation. **J. V. de Assis:** Investigation; writing – original draft; methodology; formal analysis; validation; visualization; writing – review and editing; data curation. **A. J. S. de Souza:** Data curation; supervision; formal analysis; project administration; methodology; validation; visualization; writing – review and editing; writing – original draft; investigation; conceptualization.

